# Comparing Cytoreductive Nephrectomy with Tumor Thrombectomy Between Open, Laparoscopic, and Robotic Approaches

**DOI:** 10.3390/cancers17213490

**Published:** 2025-10-30

**Authors:** Maxwell Sandberg, Gregory Russell, Phillip Krol, Mitchell Hayes, Randall Bissette, Reuben Ben David, Kartik Patel, Brejjette Aljabi, Seok-Soon Byun, Oscar Rodriguez Faba, Patricio Garcia Marchinena, Thiago Mourao, Gaetano Ciancio, Charles C. Peyton, Rafael Zanotti, Philippe E. Spiess, Reza Mehrazin, Soroush Rais-Bahrami, Diego Abreu, Stenio de Cassio Zequi, Alejandro R. Rodriguez

**Affiliations:** 1 Department of Urology, Wake Forest University School of Medicine, Winston Salem, NC 27157, USA; 2 Department of Biostatistics, Wake Forest University School of Medicine, Winston Salem, NC 27157, USA; gregory.b.russell@advocatehealth.org; 3 Department of Genitourinary Oncology, H. Lee Moffitt Cancer Center & Research Institute, Tampa Bay, FL 33601, USA; 4 Department of Urology, Icahn School of Medicine at Mount Sinai, New York, NY 10031, USA; 5 Department of Urology, University of Alabama Birmingham Medical Center, Birmingham, AL 35233, USA; 6 Department of Urology, Seoul National University Bundang Medical Center, Seoul 03080, Republic of Korea; 7 Department of Urology, Puigvert Foundation, 08025 Barcelona, Spain; 8 Department of Urology, Hospital Italiano, Buenos Aires C1181ACH, Argentina; 9 Department of Urology, A.C. Camargo Cancer Institute, Sao Paulo 01509-010, Brazil; 10 Department of Urology and Transplant Surgery, University of Miami Miller School of Medicine, Miami, FL 33131, USA; 11 Comprehensive Cancer Center, Atrium Health Wake Forest Baptist Medical Center, Winston Salem, NC 27157, USA; 12 Department of Urology, Hospital Pasteur, Montevideo 11600, Uruguay

**Keywords:** cytoreductive, tumor thrombus, operative approach

## Abstract

**Simple Summary:**

This study analyzes operative approach to cytoreductive nephrectomy with tumor thrombectomy, comparing open, laparoscopic, and robotic surgical techniques. It utilizes data from the Intercontinental Collaboration on Renal Cell Carcinoma database, which pulls patients from different continents or countries (North America, Central/South America, South Korea, and Spain), where all patients had a diagnosis of renal cell carcinoma with tumor thrombus. The specific focus was on overall survival, cancer-specific survival, and progression-free survival. No difference in any of the survival parameters studied was identified by an operative approach. The surgical approach to cytoreductive nephrectomy with tumor thrombectomy can be through open, laparoscopic, or robotic choice, and surgeon comfort and patient preference should weigh heavily in the decision making.

**Abstract:**

**Background/Objectives:** For surgical candidates with metastatic renal cell carcinoma with a tumor thrombus (mRCC-TT), surgery is cytoreductive nephrectomy with tumor thrombectomy (CN-TT). This is carried out through an open (OCN-TT), laparoscopic (LCN-TT), or robotic (RCN-TT) approach. The purpose of this study was to compare survival outcomes to CN-TT by operative approach. **Methods**: This was a retrospective analysis of all patients with a diagnosis of mRCC-TT, who underwent CN-TT from a multi-institutional database from 1999–2024. Metastatic locations were qualified as either lung, bone, brain, liver, retroperitoneum, adrenal, paraaortic nodes, or other nodes. Progression was defined as radiographic evidence of recurrence or metastasis not seen on imaging prior to CN-TT. Progression locations were all metastatic locales previously noted plus the nephrectomy bed. Overall survival (OS), cancer-specific survival (CSS), and progression-free survival (PFS) were calculated. Comparisons were performed between OCN-TT, LCN-TT, and RCN-TT. **Results**: A total of 131 patients were included in the analysis (97 OCN-TT, 25 LCN-TT, and 9 RCN-TT). The TT level was not different (*p*-value > 0.05) by approach (*p*-value > 0.05). Preoperative tumor size, final pathologic tumor subtype, and postoperative tumor size were equivalent between the three surgical approaches (*p*-value > 0.05). Rates of progression were equivalent as were all locations of disease progression in the study (*p*-value > 0.05). Median OS was 1.6 years in OCN-TT, 1.5 years in LCN-TT, and 2.5 years in RCN-TT (*p*-value = 0.42). Median CSS was 2.1 years in OCN-TT, 3 years in LCN-TT, and 2.5 years in RCN-TT (*p*-value = 0.86). PFS was 0.8 years in OCN-TT, 1.2 years in LCN-TT, and 1.2 years in RNC-TT (*p*-value = 0.76). **Conclusions**: The operative approach does not affect survival outcomes for CN-TT. Surgeon comfort and patient preference should weigh heavily in operative decision making.

## 1. Introduction

For surgical candidates with metastatic renal cell carcinoma with a tumor thrombus (mRCC-TT) gold standard of treatment in most instances remains cytoreductive nephrectomy with tumor thrombectomy (CN-TT). Operative techniques, training, and resources vary significantly across the globe [1,2]. CN-TT can be carried out through an open (OCN-TT) approach, laparoscopic (LCN-TT) approach, or robotic (RCN-TT) approach. These techniques are well described in the literature [3,4,5]. Prior research has either focused on CN-TT outcomes or survival, without an emphasis on operative approach.

It is fairly established that there is a high postoperative complication rate associated with CN-TT. Westesson et al. estimate postoperative complications to be upwards of 37% [6]. Regarding survival, Lenis et al. found CN-TT was associated with improved overall survival (OS) in patients with renal vein and infradiaphragmatic thrombus in comparison to patients not undergoing surgery with mRCC-TT [7]. However, it is accepted that metastatic disease at the time of radical nephrectomy with tumor thrombectomy confers worse OS [8]. Despite the information on the procedure overall, the choice of OCN-TT, LCN-TT, and RCN-TT and its effect on procedural outcomes remains poorly understood.

Resources for surgery vary significantly across the globe. Importantly, access to surgical robots and laparoscopic instruments are not equitable, with these surgical tools mainly used in higher income countries [9,10]. In the past, our research group, the Intercontinental Collaboration on Renal Cell Carcinoma (ICORCC), has published on overall operative approach comparisons for RCC-TT, without focusing on CN-TT specifically [1]. However, given that there is estimated to be 29–56% of patients metastatic at diagnosis, focusing on CN-TT is worthwhile [2,5,11]. Moreover, as the literature on surgical approach comparisons to CN-TT is sparse, this paper can be timely. The purpose of this analysis was to compare survival outcomes between patients who underwent CN-TT through an open approach, laparoscopic, or robotic approach from the ICORCC database.

## 2. Materials and Methods

This was a retrospective analysis of all patients with a diagnosis of mRCC-TT who underwent CN-TT from the ICORCC database from 1999–2024. ICORCC is a multi-institutional and multi-continental database that focuses its research on RCC-TT and pulls patients from North America, Central/South America, Spain, and South Korea. A total of nine institutions participated in the collaborative effort. All patients in this study required radiographic confirmation of mRCC-TT through either magnetic resonance imaging (MRI) or computerized tomography (CT). All patients underwent CN-TT through either OCN-TT, LCN-TT, or RCN-TT. Not every participating institution had a robotic program, as two institutions had no robotic access. The seven institutions that did have surgical robots started their robotic programs between approximately 2006–2008. Preoperative demographic variables collected like age, gender, race, body mass index (BMI), comorbidities, symptoms at presentation, International Metastatic Disease Consortium (IMDC) scores, preoperative tumor size, and TT level based on the Mayo Clinic classification system were documented [12]. Systemic symptoms were defined as fever, weight loss, night sweats, or paraneoplastic syndromes. Perioperative variables were surgical approach, lymph node (LN) dissection, LN yield, LN positivity rates, operative time, length of stay (LOS), postoperative tumor size, renal vein margin positivity, and soft tissue margin positivity. Complication rates and blood loss or transfusion data were not readily available in the database and so were not included. Postoperative outcomes were survival, including OS, cancer-specific survival (CSS), and progression-free survival (PFS). Metastatic locations were qualified as either lung, bone, brain, liver, retroperitoneum (not including paraaortic nodes), adrenal, paraaortic nodes, or other nodes. Progression was defined as radiographic evidence of recurrence or metastasis not seen on MRI or CT scan carried out prior to CN-TT. Progression locations were all metastatic locales previously noted plus the nephrectomy bed. Recurrence at the nephrectomy bed was either at the site of kidney removal and/or the IVC. Comparisons were performed between OCN-TT, LCN-TT, and RCN-TT using analysis of variance and chi-squared test. The Kaplan-Meier survival analysis with the log-rank test was also performed to compare OS, CSS, and PFS by approach to CN-TT. Statistical analysis was performed using a combination of SPSS Statistics Version 28 (Armonk, NY, USA) and SAS Statistics Version 9.4 (Cary, NC, USA)

## 3. Results

A total of 131 patients were included in the analysis (97 OCN-TT, 25 LCN-TT, and 9 RCN-TT; Table 1). The initial mean number of metastatic sites at the time of CN-TT was two, and equal between OCN-TT, LCN-TT, and RCN-TT (*p*-value > 0.05). Gender did not differ by operative approach (*p*-value > 0.05). Race was significantly different, and LCN-TT patients had a greater proportion of Hispanic or Latino patients (84%; *p*-value < 0.001) relative to OCN-TT (5%) and RCN-TT (22%). The body mass and Charlson Comorbidity indices were not significantly different between OCN-TT, LCN-TT, and RCN-TT (*p*-value > 0.05). The IMDC scores did not differ significantly by approach (*p*-value > 0.05). Presence of systemic symptoms did not differ by approach (*p*-value > 0.05). The TT level was also not different (*p*-value > 0.05) nor laterality of the procedure (*p*-value > 0.05). Preoperative systemic therapy was not different by approach nor was administration of postoperative systemic therapy (*p*-value > 0.05). Tumor stage, tumor grade, proportion of sarcomatoid features, proportion of rhabdoid features, renal vein margin positivity, soft tissue margin positivity, LN dissection rates, LN yield, and LN positivity rates were equivalent by approach (*p*-value > 0.05). Tumor necrosis was more common in LCN-TT (60%) and OCN-TT (74%) compared to RCN-TT (44%; *p*-value = 0.027). Tumor subtype and postoperative tumor size were equivalent between the three surgical approaches (*p*-value > 0.05). Proportion of LN dissections, LN yield, and LN positivity were equivalent between OCN-TT, LCN-TT, and RCN-TT (*p*-value > 0.05). Both renal vein margin positivity and soft tissue margin positivity were equivalent (*p*-value > 0.05). There was a greater proportion of retroperitoneal metastasis in (48%; *p*-value = 0.026) patients with LCN-TT compared to patients with OCN-TT (20%) and RCN-TT (11%). Rates of progression were equivalent as were all the locations of progression in the study (*p*-value > 0.05). The patient with a nephrectomy bed recurrence was in the IVC. Proportion of both death and cancer-specific death was equivalent in the analysis (*p*-value > 0.05).

**Figure 1:**
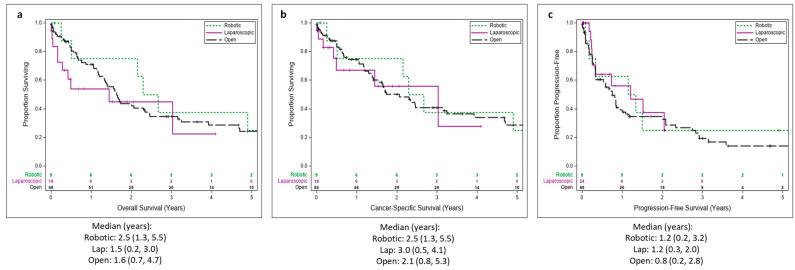
**Kaplan-Meier Survival Analysis.** Overall survival (**a**), cancer-specific survival (**b**), and progression-free survival (**c**) comparisons between open, laparoscopic, and robotic approaches. The log-rank test *p*-values are provided along with number at risk tables. Median survival with interquartile ranges is also shown below each graph.

Median OS was 1.6 [Interquartile range (IQR) 0.7–4.7) years in OCN-TT, 1.5 (IQR 0.2–3) years in LCN-TT, and 2.5 (IQR 1.3–5.5) years in RCN-TT (*p*-value = 0.42; Figure 1a). Median CSS was 2.1 (IQR 0.8–5.3) years in OCN-TT, 3 (0.5–4.1) years in LCN-TT, and 2.5 (IQR 1.3–5.5) years in RCN-TT (*p*-value = 0.86; Figure 1b). PFS was 0.8 (IQR 0.2–2.8) years in OCN-TT, 1.2 (IQR 0.3–2) years in LCN-TT, and 1.2 (0.2–3.2) years in RCN-TT (*p*-value = 0.76; Figure 1c).

## 4. Discussion

In this ICORCC database study, a comparison of CN-TT was performed based on operative approach between OCN-TT, LCN-TT, and RCN-TT. The study’s primary objective was to assess the effect of operative approach on OS, CSS, and PFS. No significant differences were identified on any survival parameter studied, nor in the location of progression after CN-TT. CN is a controversial topic. Fairly strong evidence exists that CN-TT does improve OS compared to patients who do not undergo the procedure [7,13,14]. Whether CN-TT improves CSS or PFS remains uncertain. In addition to surgical approach itself, systemic therapy has changed the management landscape of mRCC-TT. Recent clinical trials and reviews have shown that neoadjuvant systemic therapy in patients with RCC-TT can downstage TT level [15,16,17]. Additionally, ICORCC has demonstrated potential survival benefits to preoperative systemic therapy prior to CN-TT [18]. Despite this progress in the domain of CN-TT, there is a paucity of studies on operative approach.

CN is a controversial topic. Initial dogma in urologic oncology was that CN should be performed when feasible as it provided a survival benefit. Initial studies showed promise that CN followed by immunotherapy prolonged survival and possibly delayed progression rather than immunotherapy alone in RCC [19,20]. Over time, systemic therapy was revolutionized, with improved efficacy, dosing, and treatment response though [21,22,23,24]. New landmark studies with the advent of systemic therapy changed the practice landscape. These included trials such as CARMENA and SURTIME, which failed to demonstrate meaningful survival benefits with CN relative to systemic therapy alone [25,26]. Additionally, other cohort studies seemed to back this claim [27]. In mainstream practice, there is still a role for CN, but it is not as widely employed as it once was. Regarding CN-TT, data is much less robust.

Prior research on operative approach to radical nephrectomy with tumor thrombectomy (RN-TT) has indicated that operative approach plays minimal role in outcomes. Rose et al. did not focus on CN-TT specifically, but found that no difference in OS existed between 27 patients undergoing open RN-TT compared to 24 patients undergoing robotic RN-TT [28]. Similarly, Zhang et al. found no differences in OS nor CSS by operative approach (open, laparoscopic, and robotic) to RN-TT [29]. Likewise, in comparing OS and CSS, operative approach had no interaction with these outcomes. Ganeshappa et al. compared laparoscopic CN to open CN, not focusing on patients with a TT, and saw that a laparoscopic approach had lower blood loss and shorter LOS. In this analysis, no difference in LOS was noted between OCN-TT, LCN-TT, nor RCN-TT. Garg et al. compared 329 patients with RCN-TT to 1046 patients with OCN-TT, without focusing on CN-TT, finding a robotic approach had a lower blood transfusion rate regardless of the TT level [30]. Interestingly, prior research from ICORCC noted that RCN-TT may be associated with inferior metastasis-free survival [1]. However, all patients in this study had synchronous metastasis at diagnosis, making this data point less relevant. Further, no difference in PFS was identified between OCN-TT, LCN-TT, and RCN-TT. One point worth discussion is the high rate of brain metastases as a progression site. However, rates of brain metastases in RCC have a broad range, and strong evidence shows rates like our own data presented in this analysis [31,32]. Moreover, rates of progression were high in the study, regardless of operative approach. Ultimately, we feel it is not unexpected that OS, CSS, and PFS would not differ by approach to surgery. OCN-TT, LCN-TT, and RCN-TT all provide adequate surgical techniques for tumor and TT removal, and we demonstrate minimally invasive approaches to RN-TT do not compromise oncologic control. RCC-TT poses a unique challenge as there is a risk of sudden death from tumor embolization, and part of the justification to perform CN-TT in metastatic patients is to eradicate this risk. Additionally, CN-TT aims to reduce tumor burden and reduce tumor cells potentially resistant to any systemic therapy administration [28]. Macroscopic metastatic disease and its impact on disease progression is unlikely to change significantly though, and we feel these results provide sound justification to perform the surgery open, laparoscopic, or robotic.

Rates of renal vein margin positivity and soft tissue were both high in the study, and one might expect a greater rate of local recurrence given these results. However, not all patients had complete pathologic data available on these parameters, so it is possible that the true rates of renal vein and soft tissue margin positivity are lower. Further, prior research on RCC-TT has shown that vascular wall margin positivity has poor association with survival outcomes [33]. Additionally, the rates of soft tissue margin positivity are exactly within previous publication ranges for RCC-TT [34].

Our equivalent survival outcomes by operative approach did not appear to be influenced by other confounding patient factors based on univariable analysis. This includes the TT level, tumor grade, tumor stage, tumor size, and preoperative metastatic location. Nevertheless, there are multiple limitations to this study worth acknowledgment. While the multi-institutional nature of the study design is a strength, the operative technique was not standardized across the study. We cannot ensure that every RN-TT case was accounted for, however each participating institution performed rigorous chart review with multiple researchers examining charts at each study site, to minimize the risk of missing cases. Further, the retrospective nature of the analysis opens itself to selection bias. Particularly, we did not have access to the decision making behind why certain operative approaches were selected. There was not an equivalent proportion of OCN-TT, LCN-TT, and RCN-TT either. The TT level was not equally distributed across the three surgical approaches, as higher level TT trended towards being performed open. This could bias the study, and we recognize the survival results discussed may be more applicable to lower-level TT only. Moreover, the full complement of postoperative complications and blood loss or transfusion data was not available, and so we cannot comment on complication rates between OCN-TT, LCN-TT, and RCN-TT. The three patient populations also had multiple demographic/pathologic differences, notably a greater proportion of tumor necrosis in OCN-TT and LCN-TT patients along with worse IMDC scores in the OCN-TT patients, both of which have been linked to worse survival outcomes [35,36]. We also acknowledge the study includes patients who underwent CN-TT between 1999–2024 and thus includes patients from before the widespread use of targeted therapy and before the widespread use of robot-assisted procedures. It is very probable that the survival rates from before and after the widespread introduction of targeted therapy differ significantly, which has been demonstrated in previous publications, and this bias may favor robot-assisted approach [18].

## 5. Conclusions

In this series, we present one of the largest reports of CN-TT in the literature to date, coming from the ICORCC database. For the most part, the operative approach does not appear to affect outcomes for CN-TT. Importantly, OS, CSS, and PFS were not different. This is critical as we demonstrate that minimally invasive approaches do not appear to compromise survival or progression. Concurrently, for countries that lack access to surgical robots, an open approach to CN-TT also provides adequate surgical outcomes. Surgical approaches to CN-TT are adequate through an open, laparoscopic, or robotic choice, and surgeon comfort and patient preference should weigh heavily in the decision-making.

## Figures and Tables

**Table 1:** **Operative approach comparisons.** The comparison of the study population by operative approach to cytoreductive nephrectomy with tumor thrombectomy. Categorical variables are totals with percentage of the cohort in parentheses. Continuous variables are means with standard deviation in parentheses. A full complement of patient data was not available for each variable and is shown in the table with fraction of the totals available. The *p*-values for each comparison and totals or means for specific variables are provided.

Variable	OCN-TT	LCN-TT	RCN-TT	*p*-Value	Total/Mean
N	97	25	9	-	131
Female	19 (20)	5 (20)	3 (33)	0.619	27
Age (years)	62 (10)	61 (10)	65 (9)	0.638	62 (10)
Race				<0.001	
White	54/93 (58)	4 (16)	6 (67)		64
Black	8/93 (9)	0	0		8
Hispanic/Latino	5/93 (5)	21 (84)	2 (22)		28
Asian	22/93 (24)	0	1 (11)		23
Other	4/93 (4)	0	0		4
BMI	27 (6)	28 (5)	27 (8)	0.979	27 (6)
CCI	7 (3)	3 (2)	6 (3)	0.053	7 (3)
Systemic symptoms	36 (37)	12 (48)	2 (22)	0.361	50
TT level					
1	45 (46)	8 (32)	7 (78)	0.247	60
2	22 (23)	8 (32)	1 (11)		31
3	13 (13)	6 (24)	1 (11)		20
4	17 (18)	3 (12)	0		20
Right-sided	45 (46)	21 (84)	5 (56)	0.079	71
Preoperative tumor size (cm)	9 (3)	9 (3)	10 (3)	0.949	9 (3)
Operative time (minutes)	304 (142)	267 (77)	216 (62)	0.09	289 (128)
Length of stay (days)	9 (9)	6 (3)	4 (3)	0.124	8 (8)
T3a	16 (16)	8/22 (36)	4 (44)	0.337	28
T3b	50 (52)	8/22 (36)	3 (33)		61
T3c	11 (11)	4/22 (18)	1 (11)		16
T4	10 (10)	2/22 (9)	1 (11)		13
Grade				0.765	
1	2/91 (2)	0/21	0		2
2	8/91 (9)	3/21 (14)	1 (11)		12
3	42/91 (46)	5/21 (24)	4 (44)		51
4	39/91 (43)	13/21 (62)	4 (4)		56
Sarcomatoid	16 (16)	6 (24)	2 (22)	0.749	24
Rhabdoid	8 (8)	1 (4)	0	0.34	9
Necrosis	72 (74)	15 (60)	4 (44)	0.027	91
Tumor subtype					
Clear cell	74/94 (79)	19/24 (79)	8 (88)	0.651	101
Papillary	10/94 (11)	1/24 (4)	0		11
Other	10/94 (11)	4/24 (17)	1 (11)		15
Postoperative tumor size (cm)	10 (4)	10 (3)	8 (2)	0.083	10 (3)
LN dissection	52 (54)	7 (28)	3 (33)	0.059	62
LN yield	4 (5)	2 (2)	8 (10)	0.274	
LN +	1 (2)	2 (2)	0.3 (0.5)	0.552	1 (2)
Renal vein margin +	64/87 (74)	14/14 (100)	6/7 (86)	0.96	84
Soft tissue margin +	23/87 (26)	0	1/7 (14)	0.235	24
Initial number metastatic sites	1 (1)	2 (1)	3 (3)	0.024	2 (1)
Metastasis					
Lung	43 (44)	15 (60)	7 (78)	0.586	65
Bone	10 (10)	3 (12)	2 (22)	0.709	15
Liver	9 (9)	8 (24)	0	0.015	17
Brain	3 (3)	0	0	0.515	3
Retroperitoneal nodes	19 (20)	12 (48)	1 (11)	0.026	32
Paraaortic nodes	5 (5)	4 (16)	0	0.187	9
Adrenal gland	9 (9)	4 (16)	0	0.423	13
Other	11 (11)	0	2 (2)	0.092	13
IMDC				0.08	
0	8/73 (11)	2/23 (9)	0	10	10
1	20/73 (27)	6/23 (26)	3/6 (50)	29	29
2	32 (44)	3/23 (13)	2/6 (33)	37	37
3	11 (15)	10/23 (43)	1/6 (17)	22	22
4	2 (5)	1/23 (4)	0	3	3
5	0	1/23 (4)	0	1	1
Progression	62 (64)	10 (40)	6 (67)	0.085	78
Lung	12 (12)	3 (12)	2 (22)	0.832	17
Bone	9 (9)	2 (8)	2 (22)	0.734	13
Liver	6 (6)	1 (4)	1 (11)	0.76	8
Brain	11 (11)	2 (8)	1 (11)	0.638	14
Retroperitoneal nodes	6 (6)	0	1 (11)	0.322	7
Paraaortic nodes	5 (5)	1 (4)	1 (11)	0.806	7
Adrenal gland	3 (3)	0	1 (11)	0.361	4
Nephrectomy bed	1 (1)	0	0	0.794	1
Other	11 (11)	0	2 (22)	0.092	13
Preoperative systemic therapy	11 (11)	3 (12)	0	0.558	14
Postoperative systemic therapy	59 (61)	14 (56)	7 (78)	0.453	80
Dead	59 (61)	10 (40)	7 (78)	0.423	76
Cancer-specific death	49 (51)	7 (28)	7 (78)	0.114	63

## Data Availability

Data from this study is not publicly available because of patient privacy laws but is available from the corresponding author upon reasonable request in a limited, de-identified format.

## Abbreviations

The following abbreviations are used in this manuscript:

mRCC-TT	Metastatic renal cell carcinoma with a tumor thrombus
CN-TT	Cytoreductive nephrectomy with tumor thrombectomy
OCN-TT	Open cytoreductive nephrectomy with tumor thrombectomy
LCN-TT	Laparoscopic cytoreductive nephrectomy with tumor thrombectomy
RCN-TT	Robotic cytoreductive nephrectomy with tumor thrombectomy
OS	Overall survival
ICORCC	Intercontinental Collaboration on Renal Cell Carcinoma
MRI	Magnetic resonance imaging
CT	Computerized tomography
BMI	Body mass index
IMDC	International Metastatic Disease Consortium
LN	Lymph node
LOS	Length of stay
CSS	Cancer-specific survival
PFS	Progression-free survival
RN-TT	Radical nephrectomy with tumor thrombectomy
IQR	Interquartile range
